# Effects of Age and Sex on Subcortical Volumes

**DOI:** 10.3389/fnagi.2019.00259

**Published:** 2019-09-26

**Authors:** Yanpei Wang, Qinfang Xu, Jie Luo, Mingming Hu, Chenyi Zuo

**Affiliations:** ^1^State Key Laboratory of Cognitive Neuroscience and Learning, Beijing Normal University, Beijing, China; ^2^Jiangsu Provincial Key Laboratory of Special Children’s Impairment and Intervention, Nanjing Normal University of Special Education, Nanjing, China; ^3^College of Educational Science, Anhui Normal University, Wuhu, China

**Keywords:** subcortical, volume, asymmetry, sex, aging

## Abstract

**Purpose:**

In an increasingly aging society, it is of great importance to consider trajectories of subcortical volumes at different ages for understanding biological markers of aging. Thus, we investigated sex, age, and their interactions on subcortical volumes, including the basal ganglia (caudate, putamen, accumbens, and pallidum), thalamus, hippocampus, and amygdala.

**Methods:**

We analyzed the adult lifespan trajectory of subcortical volumes and asymmetries in 563 healthy subjects aged from 19 to 86 using magnetic resonance imaging (MRI) data from the publicly available 7IXI data set.

**Results:**

The sex made strong contributions to the trajectories of subcortical volumes with aging, including the right putamen, right pallidum, bilateral thalamus, hippocampus, and amygdala. The volume of the right putamen, right pallidum, and right thalamus decreased more rapidly in males than in females, and the volume of the left thalamus, bilateral hippocampus, and amygdala in males followed a quadratic model, while those in females followed a linear decline model. The asymmetries in the caudate and hippocampus showed a linear decline, and a sex and age interaction was found in the hippocampus; that is, the asymmetry in the hippocampus decreased only in the males and not in the females. Changes in the accumbens and pallidum fit quadratic trajectories, in which females increased until 39.26 years old in the accumbens asymmetry and then began to rapidly decline, and males showed a linear decline. The asymmetry in the pallidum in males and females showed a slow decreasing period until almost 45 years of age and then increased.

**Conclusion:**

The results suggest that compared with females, males have a faster decline in the volume of the right putamen, right pallidum, and right thalamus, while aging occurred later but also faster in the left thalamus, bilateral hippocampus, and amygdala. Interestingly, we found the inflection point in the thalamus, bilateral hippocampus, and amygdala volume in the quadratic model, and after this point, the volume change accelerated with aging, which may have resulted from the stronger work pressure in the middle-aged men and the low levels of testosterone in the older adults. The interaction of age and sex on individual subcortical structures provides evidence to support the impact of sex on psychopathologies associated with degenerative brain disorders in the elderly. The findings may be significant to investigate the occurrence and prevalence of degenerative brain disorders in males and females. Future studies can focus on the functional and behavioral relations with subcortical structures for preventive measures of related disorders.

## Introduction

With the development of brain imaging techniques, many studies have consistently found that the volume of the adult brain shrinks with age, which has been called atrophy, and over the adult lifespan, the most pronounced age-related effects have been observed in the frontal and temporal lobes (Raz et al., 2004b, 2005; Allen et al., 2005). Atrophy in these regions has been shown to be directly linked to cognitive or age-related deficits, but the pattern of aging in each region is not the same. Small but consistent sex differences in cognitive abilities (Jones et al., 2003) have also been observed. Furthermore, since the brain volume can be more accurately measured and used to predict and diagnose age-related neurological disorders (Dubois et al., 2007; Colliot et al., 2008), it is crucially important to understand the factors that affect alterations in volume with aging. Previous studies have mainly examined general age effects on brain morphometry (Raz et al., 2004a; Fotenos et al., 2005; Walhovd et al., 2005), but the influence of sex on brain aging remains unclear. Notably, age effects on the volume of subcortical structures related to cognitive and emotional control may be important for understanding the role of aging in age-related disorders, such as Alzheimer’s disease and psychiatric disorders.

The pathologic features of subcortical structures, such as basal ganglia (including the caudate, putamen, and pallidum), thalamus, and medial temporal structures (including the hippocampus and amygdala), have been related to cognitive and affective dysfunctions. However, men and women may have differential age trajectories regarding changes in subcortical structures. Total brain volume was, on average, almost 8–15% larger in men than in women (Ruigrok et al., 2014). Some studies focused on subcortical structures have examined sex differences; they also observed a larger volume in males; however, when the overall brain size was controlled for, the differences disappear (Rijpkema et al., 2012; Tang et al., 2013). Compared with females, males have larger gray matter (GM) volumes in the caudate (Filipek et al., 1994), but a subsequent study observed smaller GM volumes in males when brain size was controlled for Luders et al. (2009). Although the sex effects of subcortical volumes were not consistent, there are many reports on the special anatomical and functional characteristics of basal ganglia in sex-specific neuromental disorders in order to support their role in sex-specific neuropsychiatric disorders (e.g., Parkinson, ADHD, and addiction) (Bourque et al., 2009; Volkow et al., 2012; Wang et al., 2018). One study also reported a sex-specific correlation between hippocampal volume and psychometric intelligence (Colom et al., 2013).

The question of whether a substantial age × sex interaction exists has received attention in the literature. Some studies have reported age × sex interactions (Murphy et al., 1996; Coffey et al., 1998; Gur et al., 1999; Xu et al., 2000; Good et al., 2001a; Pruessner et al., 2001; Raz et al., 2004b), while others have found no age × sex interactions (Greenberg et al., 2008). The age and sex interactions observed in the previous studies have not been consistent. For example, two studies found that males have more pronounced age-related brain atrophy (Coffey et al., 1998; Gur et al., 1999), and the largest age-related effects were observed in the frontal and temporal lobes (Murphy et al., 1996; Raz et al., 2004a). Another study found age and sex interactions in the putamen, and further analysis identified a larger loss of GM in men than in women with increasing age (Murphy et al., 1996). The effects of sex on overall brain volume have been described in old age (Liu et al., 2010) and across the lifespan (Xu et al., 2000; Good et al., 2001b; Takahashi et al., 2011). In addition, large effects of age on subcortical volumes have been observed (Walhovd et al., 2011; Ziegler et al., 2012). However, only a few pioneering studies have included the effects of age and sex in subcortical atrophy models. These inconsistent results might be caused by differences in sample characteristics, scan quality, statistical procedures, and especially age ranges or different methodologies.

Various age ranges have been studied across the lifespan. For example, the age range of 18–42 years may not be suited for revealing age effects, as it would not cover volume loss in the elderly (Morrison et al., 2006). Using the same approach, Goto found hippocampal decline in males aged 60 and over and in females after menopause, which suggested a longer preservation of hippocampal integrity in males (Goto et al., 2011). Some studies have also found age-related decreases in the left basal ganglia of males but not females. Takahashi et al. (2011) divided individuals in age groups (<50 years old vs. >50 years old) and investigated age-related volume trajectories across the entire brain and reported differential effects of sex in the older age group on the relative volume of the thalamus, but importantly, they could not identify variance related to continuous age ranges. More importantly, these studies did not assess the role of sex in brain aging.

In addition, different methodologies were used in the previous studies. For example, using manual segmentation of brain structures, one study reported that the hippocampus was negatively related to age in males only, while no overall sex differences were found (Pruessner et al., 2001). A study using voxel-based morphometry (VBM) examined the interaction between sex and age trajectories on whole-brain volumes; however, they did not find differences in age trajectories for the subcortical structures in males and females (Good et al., 2001a). The VBM may not be sufficiently sensitive for detecting the different sex effects on the age trajectories in some subcortical volumes, partially because the smoothing kernel is too large. It is necessary to overcome inaccuracies in registration performance (Li et al., 2014). Thus, previous studies should be confirmed by using a more precise and objective subcortical segmentation method, which does not require spatial smoothing and specifically study the relations of age with brain volume. These studies should also consider sex-specific effects on brain atrophy in the subcortical regions across the entire adult lifespan; these effects are critical for cognitive and emotional adaptation to daily life, as well as for regional specificity with regard to molecular mechanisms. For example, the patterns of region-specific iron deposition in the basal ganglia under the influence of hormonal regulation may lead to regionally specific atrophy patterns.

Some studies have reported more age-related regional volume differences in men than in women (Xu et al., 2000; Good et al., 2001b; Pruessner et al., 2001; Raz et al., 2004b), while others have found no sex differences (Greenberg et al., 2008) or a heterogeneous pattern across different brain structures (Cowell et al., 2007). Although the interactions between age and sex are significant, the effect sizes were very small (Jäncke et al., 2015).

Thus, this study was designed to evaluate sex differences and age × sex interactions regarding subcortical volumes. We examined the existence of sex-related differences in age-dependent subcortical volume changes across the span of adulthood in a well-characterized sample of healthy individuals. We wanted to answer the following question: what is the influence of sex and age on subcortical volumes (caudate, putamen, pallidum, thalamus, hippocampus, and amygdala)? To our knowledge, only two articles using much smaller sample sizes have addressed this question (Tang et al., 2013; Li et al., 2014). Thus, our study serves as a systematic replication of these previous studies. We hypothesized that the interactions between sex and age would be specific for distinct subcortical structures, with steeper decreases with age in males, reflecting their increased vulnerability to cognitive and emotional disorders. We used subcortical GM volumes to examine age × sex interactions as well as sex influences on compartmental volumes using volBrain (Manjón and Coupe, 2016), an unbiased automated segmentation tool. These changes should represent sex-dependent age effects in the context of maturation, as well as subcortical scaled volume differences and sex-specific vulnerabilities in the process of aging.

## Materials and Methods

### Subjects

All participants from the IXI database included T1-weighted, T2-weighted, and diffusion magnetic resonance imaging (MRI) data^1^. The IXI dataset was collected from normal, healthy subjects. We only used the T1-weighted MRI data to investigate the subcortical GM volumes. The full sample consisted of 563 participants from 19 to 86 years (mean age: 48.65 ± 16.47 years; 250 males/313 females). T1-weighted images were acquired at three sites, which obtained 150, 150, and 146 sagittal slices, with the same acquisition matrix 256 × 256 and in-plane resolution of 0.93 × 0.93 × 1.2 mm^3^. The detailed information is shown in Table 1 and Supplementary Table S1.

**TABLE 1:** Sample characteristics in different age groups.

**Age group (years)**	**No. of subjects**	**No. of subjects, 1/2/3 site^a^**	**Females (%)**	**Caucasian (%)**	**Education^b^ mean (SD)**
19–29	101	41/18/42	56 (55.45)	70 (69.31)	4.44 (1.28)
30–39	99	50/22/27	39 (39.39)	75 (75.76)	4.55 (1.05)
40–49	89	53/11/25	48 (53.93)	75 (84.27)	4.03 (1.36)
50–59	99	65/3/31	61 (61.62)	81 (81.82)	3.46 (1.50)
60–69	118	70/7/41	72 (61.02)	98 (83.05)	3.55 (1.58)
70–79	49	29/6/14	34 (69.39)	44 (89.80)	3.39 (1.57)
80–86	8	6/1/1	3 (37.50)	8 (100.00)	3.50 (2.07)
Total	563	314/68/181	313 (55.60)	451 (80.11)	3.93 (1.47)

*^*a*^Samples from different scanning sites in London: Guys Hospital Philips; Institute of Psychiatry General Electric; Hammersmith Hospital. ^*b*^Education levels: 1 = no qualifications; 2 = O-levels, GCSEs, or CSE; 3 = A-levels; 4 = further education; 5 = university or polytechnic degrees. SD, standard deviation.*

### Data Preprocessing

**FIGURE 1:**
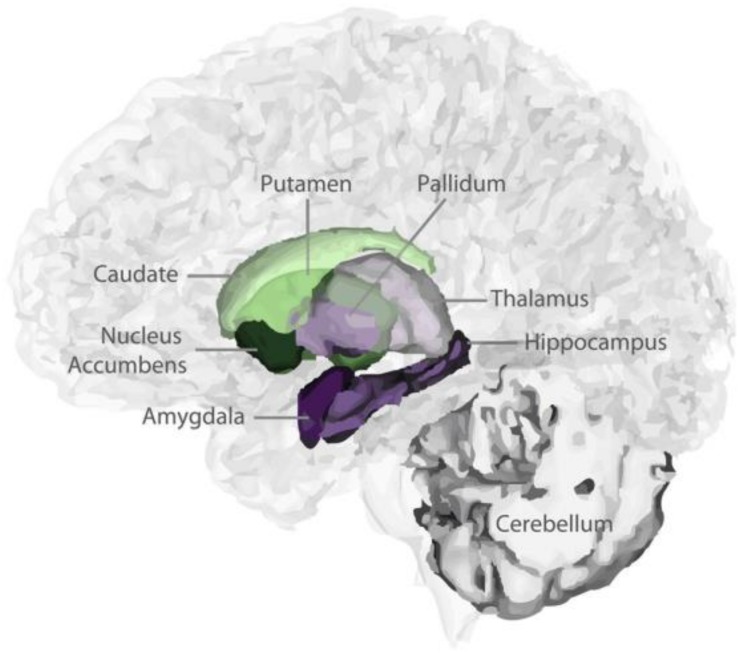
3D view of subcortical segmentations.

T1-weighted images were processed by “volBrain” (Manjón and Coupe, 2016), which is an advanced pipeline that provides automatic volumetric information from the brain MR images at different scales. The results provided the subcortical GM volumes (putamen, caudate, pallidum, thalamus, hippocampus, amygdala, and accumbens; Figure 1). The asymmetry index is calculated as the difference between right and left thickness divided by their mean (in percent) (Wang et al., 2018): Asymmetry = (left volume - right volume)/mean (left volume, right volume) × 100%. The asymmetry index indicates the degree of left lateralization.

### Statistical Analysis

All statistical analyses were conducted using SPSS 23.0 (SPSS, Chicago, IL, United States). We used linear and non-linear regressions to estimate the effects of age and sex on the subcortical volumes from the quadratic model to the linear model, including the main effects of sex, age, and the interaction of sex and age (Narvacan et al., 2017). Each subcortical volume was fitted to (i) quadratic: subcortical volume = intercept + A(sex) + B(age) + C(age^2^) + D(age × sex) + E(age^2^ × sex) + residual error; or (ii) linear: subcortical volume = intercept + A(sex) + B(age) + C(age × sex) + residual error. If the age and sex interaction was not significant, each subcortical volume was re-fit with only sex and age main effects. There are a total of four fits for each measure to choose in the model (detailed in Supplementary Tables S2–S5). The choice of the best model fit was based on two steps (Wierenga et al., 2014): First, quadratic and linear age effects were fitted on the each subcortical volume. If the quadratic age effect was not significant, we stepped down to the linear developmental model. If a measure fit a quadratic model, we would calculate the age at peak from the first derivative. Second, we investigated whether the developmental trajectories differed between genders. Statistical significance was *p* < 0.05, adjusting for the number of comparisons.

Bonferroni correction was used for multiple comparisons. To optimally balance between Type I and Type II error, we took the correlation between the dependent variables (volumes of the seven subcortical structures) into account by using the Simple Interactive Statistical Analysis Bonferroni tool^2^. Using a Bonferroni correction that treats the variables as independent (proper Bonferroni: alpha/number of tests) would lead to a too stringent correction, as the dependent variables are not obtained in independent subgroups. Subcortical volumes showed a mean correlation coefficient of *r* = 0.54, leading to an equivalent corrected alpha of 0.015. Subcortical asymmetry showed a mean correlation coefficient of *r* = 0.11, and therefore a significant level of alpha = 0.013 is equivalent to a corrected *p* < 0.05.

## Results

### Subcortical Volumes

**FIGURE 2:**
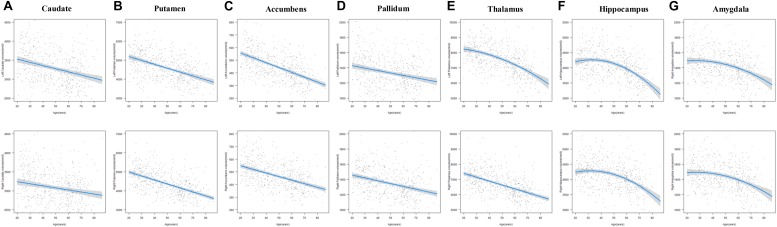
The age trajectories of left (upper layer graph) and right (lower layer graph) subcortical volumes, including the **(A)** caudate, **(B)** putamen, **(C)** accumbens, **(D)** pallidum, **(E)** thalamus, **(F)** hippocampus, and **(G)** amygdala.

The results of the regression models for each subcortical volume are shown in Figures 2, 4 and Tables 2, 4. The bilateral caudate (Figure 2A), putamen (Figure 2B), accumbens (Figure 2C), and pallidum (Figure 2D) and the right thalamus (Figure 2E) were fit with a linear model; that is, volume declined with increased age. The age × sex interactions were found in the right putamen, right pallidum, and right thalamus. Subsequent analysis (Table 4) found that males showed a strong linear decline with age compared with females in the right putamen (Figure 4B), right pallidum (Figure 4D), and right thalamus (Figure 4E). The left thalamus, bilateral hippocampus (Figure 2F), and bilateral amygdala (Figure 2G) were best fit to curves, indicating an increase and subsequent gradual decline. In a separate analysis of sex, we found that females fit a linear decline with aging, while males fit curves in the left thalamus (Figure 4E), bilateral hippocampus (Figure 4F), and bilateral amygdala (Figure 4G). The age at the peak volume of the left thalamus (24.99 years), bilateral hippocampus (left: 36.76 years; right: 36.15 years), and bilateral amygdala (left: 26.40 years; right: 32.63 years) was at approximately 30 years of age in men.

**TABLE 2:** Fitting parameters for subcortical volumes versus age within each hemisphere.

**Parameter (S.E.)**								
**Measures**	**HS**	**Best fit model**	**Intercept^a^ (×10^3^)**	**Sex (×10^2^)**	**Age**	**Age^2^ (×10*^–^*^1^)**	**Age × Sex**	**Age^2^ × Sex**
Caudate	L	Linear	3.74 (0.07)	3.22 (0.40)	−9.35(1.23)	–	–	–
	R	Linear	3.38 (0.07)	3.17 (0.39)	−6.26(1.22)	–	–	–
Putamen	L	Linear	5.66 (0.08)	4.99 (0.51)	−21.61(1.55)	–	–	–
	R	Linear	5.25 (0.09)	7.64 (1.35)	−17.82(1.76)	–	−5.89 (2.64)	–
Accumbens	L	Linear	0.63 (0.01)	0.34 (0.08)	−3.83(0.24)	–	–	–
	R	Linear	0.60 (0.01)	0.47 (0.08)	−2.82(0.23)	–	–	–
Pallidum	L	Linear	1.52 (0.03)	1.54 (0.18)	−3.86(0.55)	–	–	–
	R	Linear	1.48 (0.03)	2.75 (0.46)	−2.58(0.60)	–	−2.47 (0.89)	–
Thalamus	L	Quadratic	8.34 (0.31)	8.90 (0.69)	n.s.	−3.41 (1.38)	–	–
	R	Linear	7.70 (0.12)	10.96 (1.80)	−21.20(2.36)	–	−9.82 (3.54)	–
Hippocampus	L	Quadratic	3.85 (0.17)	3.18 (0.37)	25.27 (7.28)	−3.90 (0.74)	–	–
	R	Quadratic	3.96 (0.16)	2.64 (0.36)	20.63 (7.10)	−3.30 (0.72)	–	–
Amygdala	L	Quadratic	1.38 (0.07)	1.50 (0.15)	n.s.	−0.91 (0.31)	–	–
	R	Quadratic	1.43 (0.07)	1.56 (0.15)	n.s.	−0.88 (0.31)	–	–

*^*a*^Intercept is the extrapolated value at age zero. – = not applicable; n.s. = non-significant; HS = hemisphere; L = left; R = right. The level of significance is 0.015 after multiple comparisons correction.*

### Subcortical Asymmetries

**FIGURE 3:**
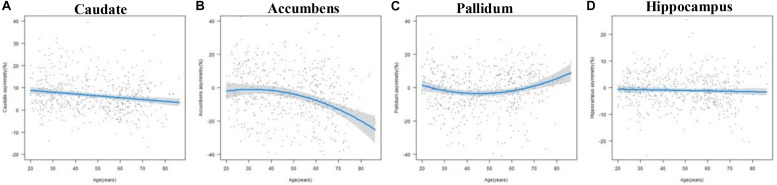
The age trajectories of subcortical asymmetries, including the **(A)** caudate, **(B)** accumbens, **(C)** pallidum, and **(D)** hippocampus.

The results of the regression models for each subcortical asymmetry are shown in Figures 3, 5 and Tables 3, 5. The caudate (Figure 3A) and hippocampus (Figure 3D) asymmetry fit a linear model, that is, a decline with aging. The age × sex interactions were found in the hippocampus. Further analysis (Table 5) found that the hippocampus asymmetry decreased with aging in females, but not in males (Figure 5D). The accumbens (Figure 3B) and pallidum (Figure 3C) asymmetry fit a quadratic model, and the accumbens showed a significant age × sex interaction (Figure 3B). Subsequent analysis (Table 5) showed that the accumbens fit a quadratic model in females (peak age: 39.26) and the linear model in males (Figure 5B), and the pallidum fit a quadratic model in males (peak age: 44.21) and females (peak age: 46.53) (Figure 5C).

**FIGURE 4:**
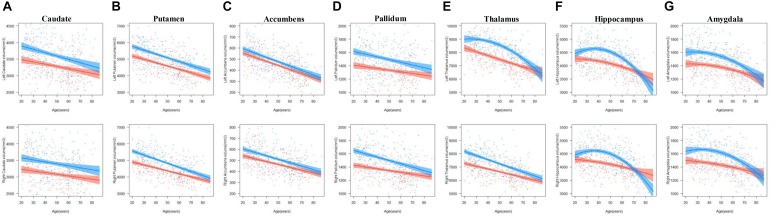
The age trajectories of left (upper layer graph) and right (lower layer graph) subcortical volumes, including the **(A)** caudate, **(B)** putamen, **(C)** accumbens, **(D)** pallidum, **(E)** thalamus, **(F)** hippocampus, and **(G)** amygdala in females (red color) and males (blue color).

**FIGURE 5:**
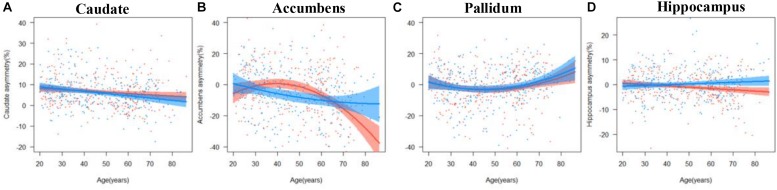
The age trajectories of subcortical asymmetries, including the **(A)** caudate, **(B)** accumbens, **(C)** pallidum, and **(D)** hippocampus in females (red color) and males (blue color).

**TABLE 3:** Fitting parameters for asymmetries of subcortical structures versus age.

**Parameter (S.E.)**							
**Measures**	**Best fit model**	**Intercept^a^ (×10)**	**Sex**	**Age (×10*^–^*^1^)**	**Age^2^ (×10*^–^*^2^)**	**Age × Sex (×10*^–^*^1^)**	**Age^2^ × Sex (×10*^–^*^2^)**
Caudate	Linear	1.05 (0.12)	n.s.	−0.83(0.21)	−	−	−
Putamen	None	−	−	−	−	−	−
Accumbens	Quadratic	−2.59(0.97)	n.s.	13.58 (4.25)	−1.73(0.43)	−18.50(6.27)	2.01 (0.63)
Pallidum	Quadratic	*n*.*s*.	n.s.	−6.83(2.11)	0.75 (0.21)	−	−
Thalamus	None	−	−	−	−	−	
Hippocampus	Linear	*n*.*s*.	n.s.	−0.53(0.22)	−	0.85 (0.33)	−
Amygdala	None	−	−	−	−	−	−

*^*a*^Intercept is the extrapolated value at age zero. – = not applicable; n.s. = non-significant. The level of significance is 0.013 after multiple comparisons correction.*

**TABLE 4:** Fitting parameters for subcortical volumes versus age within each hemisphere in females and males.

**Parameter (S.E.)**									
**Measures**	**HS**	**Females**				**Males**			
		**Best fit model**	**Intercept^a^ (×10^3^)**	**Age (×10)**	**Age^2^ (×10*^–^*^1^)**	**Best fit model**	**Intercept^a^ (×10^3^)**	**Age (×10)**	**Age^2^ (×10*^–^*^1^)**
Caudate	L	Linear	3.67 (0.08)	−0.81 (0.15)	–	Linear	4.14 (0.10)	−1.11 (0.20)	–
	R	Linear	3.37 (0.08)	−0.59 (0.15)	–	Linear	3.73 (0.10)	−0.68 (0.20)	–
Putamen	L	Linear	5.90 (0.10)	−2.04 (0.20)	–	Linear	6.23 (0.12)	−2.31 (0.25)	–
	R	Linear	5.25 (0.09)	−1.78 (0.17)	–	Linear	6.06 (0.10)	−2.48 (0.20)	–
Accumbens	L	Linear	0.63 (0.02)	−0.37 (0.03)	–	Linear	0.67 (0.02)	−0.39 (0.04)	–
	R	Linear	0.59 (0.02)	−0.26 (0.03)	–	Linear	0.67 (0.02)	−0.31 (0.04)	–
Pallidum	L	Linear	1.47 (0.04)	−0.28 (0.07)	–	Linear	1.74 (0.04)	−0.52 (0.08)	–
	R	Linear	1.48 (0.03)	−0.26 (0.06)	–	Linear	1.75 (0.03)	−0.51 (0.07)	–
Thalamus	L	Linear	8.91 (0.14)	−2.87 (0.27)	–	Quadratic	8.55 (0.47)	n.s.	−7.41 (2.08)
	R	Linear	7.70 (0.12)	−2.12 (0.23)	–	Linear	8.79 (0.13)	−3.10 (0.27)	–
Hippocampus	L	Linear	4.56 (0.07)	−1.04 (0.14)	–	Quadratic	3.76 (0.26)	4.73 (1.14)	−6.44 (1.15)
	R	Linear	4.50 (0.07)	−0.84 (0.13)	–	Quadratic	3.82 (0.25)	4.39 (1.09)	−6.07 (1.10)
Amygdala	L	Linear	1.53 (0.03)	−0.34 (0.06)	–	Quadratic	1.52 (0.10)	n.s.	−1.22 (0.45)
	R	Linear	1.57 (0.03)	−0.31 (0.06)	–	Quadratic	1.50 (0.11)	n.s.	−1.55 (0.48)

*^*a*^Intercept is the extrapolated value at age zero. – = not applicable; n.s. = non-significant. The level of significance is 0.015 after multiple comparisons correction.*

**TABLE 5:** Fitting parameters for asymmetries of subcortical structures versus age in females and males.

	**Parameter (*p*-value)**							
**Measures**	**Females**				**Males**			
	**Best fit model**	**Intercept^a^ (×10)**	**Age**	**Age^2^ (×10)**	**Best fit model**	**Intercept^a^ (×10)**	**Age**	**Age^2^ (×10)**
Caudate	Linear	0.94 (0.16)	−0.06 (0.03)	–	Linear	1.11 (0.14)	−0.11 (0.03)	–
Putamen	None	–	–	–	None	–	–	–
Accumbens	Quadratic	−2.59 (1.01)	1.36 (0.44)	−0.17 (0.04)	Linear	n.s.	−0.22 (0.07)	
Pallidum	Quadratic	n.s.	n.s.	0.07 (0.03)	Quadratic	n.s.	n.s.	0.08 (0.03)
Thalamus	None	–	–	–	None	–	–	–
Hippocampus	Linear	n.s.	−0.05 (0.02)	–	None	–	–	–
Amygdala	None	–	–	–	None	–	–	–

*^*a*^Intercept is the extrapolated value at age zero. – = not applicable; n.s. = non-significant. The level of significance is 0.013 after multiple comparisons correction.*

## Discussion

In the present study, we studied the association between age and subcortical volume in regions that are important for cognitive and emotional adaptations to daily life across the lifespan. We explicitly examined these relationships in males and females.

Obvious contributions of age were observed in the basal ganglia structures (i.e., caudate, putamen, accumbens, and pallidum), thalamus, and medial temporal structures (hippocampus and amygdala); in detail, the basal ganglia and right thalamus show a linear decline with aging, and the left thalamus and medial temporal structures fit a quadratic model. We observed strong contributions of sex on the volume trajectories of subcortical structures with aging, including the right putamen, right pallidum, bilateral thalamus, bilateral hippocampus, and bilateral amygdala. The volume of the right putamen, right pallidum, and right thalamus declined more steeply in males than in females, and the left thalamus, bilateral hippocampus, and amygdala volumes followed a quadratic model in males, while a linear decrease model fit in females. We also analyzed the asymmetries in the subcortical structures. The caudate and hippocampus displayed a linear decrease, and the sex and age interaction was found in the hippocampus; that is, the asymmetry decreased in the hippocampus only among females and not among males. The accumbens and pallidum fit quadratic trajectories: the accumbens asymmetry in females increased until 39.26 years old and then began to rapidly decrease; males decreased across all ages in the accumbens; and the pallidum asymmetry in males and females gradually decreased until almost 45 years and then gradually increased. The asymmetry results suggested that the caudate showed left lateralization, which continued to decrease until approaching zero; the pallidum gradually showed left lateralization, which continued to increase; the accumbens gradually showed right lateralization that continued to increase, especially in females; and the hippocampus showed right lateralization and increased with age only in males.

Within the basal ganglia, a group of nuclei (the caudate, putamen, accumbens, and pallidum) involved in cognitive, emotional, and motor behavior (Alexander et al., 1986) all atrophied with aging. Previous studies have found that the basal ganglia reached a peak volume before 20 years of age, while the youngest participant in our sample was 20. Therefore, the basal ganglia had already been going through a period of atrophy. The effect of sex on the volume of subcortical structures might play a key role, since the basal ganglia possess a high density of sex steroid receptors (Taber et al., 2001). During this period, the interaction of age and sex suggested that males had a more pronounced age progression in the right putamen and right pallidum. Our results echoed a previous study reporting an age-related decrease in the basal ganglia volume over a similar age range (Goodro et al., 2012). The age-related atrophy of the basal ganglia was confirmed by previous reports (Goodro et al., 2012), and the brain volume was more vulnerable to shrinkage with age in men than in women, although this effect was not found in basal ganglia volume. For example, Li et al. (2014) found no sex and age interaction in the striatum using a similar sample. In the present study, we used an automatic and reliable quantitative analysis approach to address volumetric alterations, especially in regions with low tissue contrast (Manjón and Coupe, 2016). Compared with females, males have been found to have worse performance in a sustained reaction task and a visuospatial learning and planning task, especially at older ages (Clark et al., 2006; Proust-Lima et al., 2008). In contrast, several studies did not report age effects, sex effects, or differential aging effects on cognitive performance in males and females (Silver et al., 2011; Kavé et al., 2012), which suggests that the steeper decrease in the basal ganglia volume may be more closely associated with male climacteric emotional/somatosensory abnormalities, including late-life depression, anxiety, and sleep problems. However, understanding the direct relations among relative volumes, task performance, and emotional and somatosensory disorders needs further investigation.

Although MRI research cannot identify the mechanism leading to volume reduction, when combined with advanced software technology, these studies are able to provide Supplementary Information for the potential mechanism of age-related volume reductions in the basal ganglia. The results have certain significance for age-related striatal atrophy. The volume analysis of the striate nucleus is helpful for evaluating neurodegenerative diseases. In addition, age-related reductions are more rapid among males; this accelerated decrease with age in males may be the reason why males are more likely to have dyskinesia. Moreover, this mechanism may contribute to the disruption of motor and cognitive function in elderly individuals, which is a symptom of age-related striatal atrophy.

In the thalamus, our results echoed previous studies (Van Der Werf et al., 2001) reporting that thalamic volume reductions correlated with age. However, the aging trajectories for males and females were different in the left and right thalamus. The left thalamic volume followed a quadratic model with age, while the right volume showed a linear decline with age. The sex and age interaction in the right thalamus showed that males had a more pronounced age regression than females, while the left thalamic volume in males increased until 25 years of age, then decreased with age; thalamic volume always decreased with age in females. Consistent with the right thalamus, after 25 years of age, the males showed a steeper reduction than females. A previous study (Raznahan et al., 2014) found that the left thalamic volume reached a peak later in males than in females, which explains why males show a quadratic trajectory. After the peak volume, both the left and right thalamus atrophied faster in males than in females. Our results were consistent with a previous study that found an age-related decrease in the volume of the thalamus over a similar age range (35–60 years) (Goodro et al., 2012), and they found that the age effect on the thalamic volume was 3% per decade for the middle-aged group and less than 1% in the older group. These results were also confirmed by another study, which reported a lower rate of thalamic volume reduction in elderly compared with middle-aged samples. One study also found that thalamic volume showed a significant correlation with age (Cherubini et al., 2009). Another study found that the structure–function connectivity between the thalamus and the orbitofrontal and frontal areas made a major contribution to age estimation, which played a key role in the process of healthy aging (Bonifazi et al., 2017). Hughes et al. (2012) suggested that age primarily affected thalamic nuclei connecting to the frontal cortex. With the Stroop test, they found that the volume of the thalamo-frontal projections was associated with executive functions. The thalamus, with its cortical, subcortical, and cerebellar connections, is a very important node in the networks that support cognitive functions known to decline in normal aging, including component processes of memory and attention (Fama and Sullivan, 2015), working memory (Charlton et al., 2010), processing speed (Van Der Werf et al., 2001), and error awareness (Peterburs et al., 2011). The sex differences in thalamic atrophy may result from sex differences in the neurotransmitter systems, as reported from animal models and clinical human data. Zubieta et al. (1999) examined age- and sex-associated variations in mu-opioid receptor binding. They observed sex × age interactions in the thalamus. Overall, women showed higher mu-opioid receptor binding values than men, although these values were reduced during postmenopause in women. Furthermore, they found that receptor binding may be related to atrophy because when there was a correction for atrophic changes, women and men did not show such differences (Zubieta et al., 1999). However, whether receptor binding influences atrophy or atrophy influences receptor binding remains unclear. To date, few papers have focused on sex differences in thalamic aging, and they did not find sex and age × sex interaction effects. We thought that the difference in sex and age in the thalamus may be a consequence of the differences in the methods and the relatively small sample size. In most studies, the VBM method was used to identify the sex differences in the subcortical nuclei. Although VBM is an excellent tool for the study of focal GM density differences, this voxel-based optimization method may not be sensitive enough to detect the sex effects on the volume of thalamus in the context of a reduced volume of subcortical structures. Further studies of the volume of the thalamus should take into account the numerous subnuclei in the structure, as the structure and function of the subnuclei are very different (Metzger et al., 2010).

The medial temporal structures included the hippocampus and amygdala among the subcortical structures. The changes in volume of the hippocampus and amygdala followed quadratic trajectories with aging. No sex and aging interaction was found; however, males and females followed different trajectories, with the former fitting the quadratic model and the latter showing a linear decline with aging, in the bilateral hippocampus and amygdala. In the hippocampus, males reached the peak volume at approximately 36 years old (left: 36.76; right: 36.15), which then began to decrease, and the atrophy speed became more rapid and was faster than that in females at approximately 70 years old. The hippocampus has been previously characterized by a non-linear pattern of estimated volume changes through adulthood. This pattern might be explained by a prolonged phase of development (Østby et al., 2009), a longer stable period, and critically, an accelerated volume loss starting around age 50, with a more robust negative relationship above 60 years of age (Fjell and Walhovd, 2010; Fjell et al., 2010). In our results, we found that females showed atrophy earlier, but in a previous longitudinal analysis, the hippocampus showed the fastest rate of volume reduction among the subcortical structures (Fjell et al., 2013). Changes in brain volume constitute a dynamic process with a large number of potential influencing factors, which should ideally be monitored by using longitudinal methods with high density assessment. These more complex and sophisticated analytical methods, as well as generating a large amount of data, can provide a deeper understanding of particular issues (Raz and Lindenberger, 2011). Within the age range of 19 to 86 years, we found an accelerated volume loss with aging. Strikingly, this process proved to occur earlier in females, while it occurred at a later time but with a faster pace in males. This may be closely related to the differential effects of stress on affected memory functions in males and females and stress-impaired spatial memory in females but not in males (Guenzel et al., 2014). The differences may be due to sex-dependent effects of stress on memory, particularly hippocampus-dependent memory, and stress may enhance hippocampus-dependent memory in males but not in females (Andreano and Cahill, 2006). Sex differences in the impact of stress on hippocampus-dependent memory are related to different concentrations of sex hormones, which are known to affect the response to stress (Galea et al., 2014). For example, hippocampal long-term potentiation patterns vary across the estrous cycle, and estradiol enhances hippocampal long-term potentiation in males (Foy et al., 2004). We also found that the hippocampal volume in females decreased more quickly than males after 50 years of age, which may be due to decrease in hormone levels. These findings emphasize that future studies need to measure or experimentally manipulate sex hormone concentrations to assess their role in the sex-dependent effects of acute stress on memory dependent on the hippocampus.

In addition to the hippocampus, another important structure among the medial temporal lobe (MTL) structures was the amygdala. Many studies have found the role of the amygdala in emotional memory, emotional facial expression recognition, and emotional auditory recognition (Adolphs et al., 1994; Cahill et al., 1996; Scott et al., 1997), which also together with hippocampus moderate learning and memory. We found that the volume changes in the bilateral amygdala fit quadratic models. Further analysis found that the trajectories were different in females and males; the former decreases with aging, while the latter follow quadratic models and reached peaks at 26.40 and 32.63 years of age in the left and right hemisphere, respectively. Our results showed that females were more easily impaired, which may be the result of female-specific biological vulnerability and stress-related environmental factors (Kornstein et al., 2000). Epidemiological studies have consistently demonstrated sex differences in the prevalence of depression, e.g., two times higher for women than men (Weissman et al., 1993). The amygdala is usually considered to be the basic structure related to emotional evaluation. In the study of neuromental disorders, the amygdala is often located as the reference area. The amygdala plays a critical role in the etiology of depression, and a recent neuroimaging study found that individuals with depressive disorder displayed significantly decreased GM volume in the amygdala (Yang et al., 2017) and a meta-analysis also found that patients with higher Hamilton Depression Rating Scale scores were significantly more likely to present reduced GM volumes in the right amygdala (Zhang et al., 2016). We also found that males have a faster rate of atrophy in the amygdala after approximately 60 years. Although an understanding of the changes in the volume of the amygdala has not yet been clarified, the changes in hormone levels and the ensuing sensitivity of the brain to hormone effects are the most certain. Sex hormones have been found to severely affect the maturation of areas of amygdala. For instance, it has been confirmed that higher circulating testosterone levels correlated positively with amygdala volume (Scott et al., 1991). Testosterone concentrations in men decrease with increasing age, and a significant proportion of men over 60 years of age have circulating testosterone concentrations in the range conventionally considered to be hypogonadal (Harman et al., 2001). This is a reasonable explanation for the rapid rate of reductions in the volume of the amygdala in older males.

## Limitations

There are several limitations in the study. The very small sample of 80–89 (with only one subject for two sites) is really a worry; therefore, we add sensitivity analysis excluding the oldest age range. As mentioned above, the type and size of volume change may depend on the age range of the study. This study was a cross-sectional study comparing young and elderly subjects with an average age of 48.56. Therefore, the cohort effect may have affected the morphological characteristics. The nutritional status, education, health, and social interactions have basically changed within 19–86 years. At the same time, there is much evidence that these factors affect the anatomical structure of the brain (Pannacciulli et al., 2006; Taki et al., 2006). Therefore, we cannot rule out that our results were affected at least to some extent by these factors. Longitudinal research will help to control these cohort effects. However, it is almost impossible to carry out such a longitudinal MRI study that covers the age range of our study. Some evidence has suggested that cross-sectional and longitudinal samples produce similar age-related patterns (Fjell et al., 2013). However, a lack of consensus was observed across different longitudinal studies. For example, according to some longitudinal studies, the basal ganglia GM is at the highest volume in childhood (Mills et al., 2016), but according to other studies, the peak value is in adolescence (Lenroot et al., 2007). Therefore, the longitudinal or cross-sectional nature of the data is another factor that introduces variability, but it is not the only factor that explains the different results reported in the literature. In addition, a follow-up study could be conducted with a wider range of lifespan data that includes teenagers (below 19 years of age), and such a study is necessary to understand the effects of age and sex on the volume of these nuclei. In the present study, we were concerned with identifying a starting point of the aging process; perhaps we could identify the inflection point between maturing and aging in the wider range of lifespan data.

## Conclusion

In a large cross-sectional data across the adult lifespan, we examined the influence of sex and age on the volume of subcortical structures, and the interactions of age × sex. Through an aging and sex analysis, the results suggested that compared with females, males have a faster reduction in the volume of the right putamen, right pallidum, and right thalamus, while aging occurred later but also faster in the left thalamus, bilateral hippocampus, and amygdala. Interestingly, we found the inflection points based on the quadratic model for the change in volume in the thalamus, bilateral hippocampus, and amygdala volume, and after this time, rate of the volume change accelerated with aging; this may have resulted from stronger work pressures in middle-aged men and low levels of testosterone in older adults. The finding of an age and sex interaction in individual subcortical structure supports the effect of sex on psychopathology associated with reduced cognitive reserve or depression sickness, especially in degenerative encephalopathy in the elderly. Our findings are critical to the study of the incidence and prevalence of dementia and depression during aging in both men and women. Future investigations into the function and behavior of more precisely identified subcortical structures may have an impact on the prevention and treatment of related diseases.

## Data Availability Statement

Publicly available datasets were analyzed in this study. This data can be found here: http://www.brain-development.org.

## Author Contributions

YW analyzed the data and wrote the draft of the manuscript. CZ, QX, JL, and MH amended and proofread the draft of the manuscript. CZ and QX participated in the discussion and offered some good ideas. All authors reviewed the manuscript.

## Conflict of Interest

The authors declare that the research was conducted in the absence of any commercial or financial relationships that could be construed as a potential conflict of interest.

## Footnotes

1 http://www.brain-development.org

2 http://www.quantitativeskills.com/sisa/calculations/bonfer.htm
