# Supplementary material for: Effects of Age and Sex on Subcortical Volumes
Source: Front Aging Neurosci. 2019 Sep 26;11:259. doi: 10.3389/fnagi.2019.00259 (PMC6775221; doi:10.3389/fnagi.2019.00259)
Supplement: Supplementary file 1 [file Table_1.DOCX]

**Supplemental Information**

Table S1. Sample characteristics in different site ^a^

|  |  | Site | | | χ2 | p value |
| --- | --- | --- | --- | --- | --- | --- |
|  |  | Guys | IOP | HH |  |  |
| Sex | females | 175 | 44 | 94 | 3.272 | 0.195 |
|  | males | 139 | 24 | 87 |  |  |
| Ethnicity | Caucasian | 142 | 39 | 92 | 4.456 | 0.348 |
|  | Chinese | 82 | 12 | 39 |  |  |
|  | Others | 90 | 17 | 50 |  |  |
| Educationb | 1 | 146 | 25 | 87 | 2.672 | 0.263 |
|  | 2 | 168 | 43 | 94 |  |  |

^a^ Samples from different scanning sites in London: Guys = Guys Hospital Philips; IOP = Institute of Psychiatry General Electric; HH = Hammersmith Hospital. ^b^1 = below University or Polytechnic degree, 2 = University or Polytechnic degree

Table S2. Fitting parameters for subcortical volumes versus age within each hemisphere

|  |  |  | Parameter ( S.E.) | | | | | |
| --- | --- | --- | --- | --- | --- | --- | --- | --- |
| Measures | HS | Best fit model | Intercept^a^  (×10^3^) | Sex(×10^2^) | Age | Age^2^(×10^-1^) | age×sex | age^2^×sex |
| Caudate | L | Linear | 3.73(0.07) | 3.25(0.40) | -9.20(1.23) | - | - | - |
|  | R | Linear | 3.38(0.07) | 3.18(0.39) | -6.20(1.22) | - | - | - |
| Putamen | L | Linear | 5.67(0.09) | 5.02(0.52) | -21.96(1.61) | - | - | - |
|  | R | Linear | 5.25(0.09) | 7.94(1.36) | -17.69(1.77) | - | -6.64(2.67) | - |
| Accumbens | L | Linear | 0.63(0.01) | 0.33(0.08) | -3.85(0.25) | - | - | - |
|  | R | Linear | 0.61(0.01) | 0.49(0.08) | -2.87(0.24) | - | - | - |
| Pallidum | L | Linear | 1.52(0.03) | 1.58(0.18) | -3.89(0.57) | - | - | - |
|  | R | Linear | 1.49(0.03) | 2.67(0.47) | -2.82(0.61) | - | -2.29(0.93) | - |
| Thalamus | L | Quadratic | 8.07(0.34) | 8.95(0.69) | n.s. | -4.93(1.54) | - | - |
|  | R | Linear | 7.72(0.13) | 10.60(1.86) | -21.71(2.42) | - | -8.94(3.70) | - |
| Hippocampus | L | Quadratic | 3.90(0.18) | 3.36(0.37) | 22.54(7.94) | -3.58(0.82) | - | - |
|  | R | Quadratic | 4.10(0.17) | 2.89(0.35) | 12.72(7.67) | -2.40(0.79) | - | - |
| Amygdala | L | Quadratic | 1.32(0.07) | 1.52(0.15) | n.s. | -1.25(0.34) | - | - |
|  | R | Quadratic | 1.43(0.07) | 1.61(0.15) | n.s. | -0.87(0.34) | - | - |

^a^ Intercept is the extrapolated value at age zero.

– = not applicable; n.s. = non-significant; HS = Hemisphere; L = left; R = Right.

*p* <0.05, Bonferroni corrected with thresholds of 0.015 volumes

Table S3. Fitting parameters for asymmetries of subcortical structures versus age

|  |  | Parameter(S.E.) | | | | | |
| --- | --- | --- | --- | --- | --- | --- | --- |
| Measures | Best fit model | Intept^a^  (×10) | Sex | Age(×10^-1^) | Age^2^  (×10^-2^) | age×sex (×10^-1^) | age^2^×sex (×10^-2^) |
| Caudate | Linear | 1.06(0.12) | n.s. | -0.86(0.22) | - | - | - |
| Putamen | None | - | - | - | - | - | - |
| Accumbens | Quadratic | -2.80(0.99) | n.s. | 14.59(4.39) | -1.84(0.45) | -18.18(6.81) | 1.97(0.70) |
| Pallidum | Quadratic | n.s. | n.s. | -6.83 (2.34) | 0.75(0.24) | - | - |
| Thalamus | None | - | - | - | - | - |  |
| Hippocampus | Linear | n.s. | n.s. | -0.55(0.22) | - | 0.82(0.33) | - |
| Amygdala | None | - | - | - | - | - | - |

^a^ Intercept is the extrapolated value at age zero. – = not applicable; n.s. = non-significant

*p* <0.05, Bonferroni corrected with thresholds of 0.013 volumes

Table S4. Fitting parameters for subcortical volumes versus age within each hemisphere

|  |  |  | | Parameter ( S.E.) | | | | | |
| --- | --- | --- | --- | --- | --- | --- | --- | --- | --- |
| Measures | HS | Fit model | | Intercept^a^  (×10^3^) | Sex(×10^2^) | Age | Age^2^(×10^-1^) | age×sex | age^2^×  sex |
| Caudate | L | **Linear** | **3.74(0.07)** | | **3.22(0.40)** | **-9.35(1.23)** | - | - | - |
|  |  | Linear | 3.67(0.08) | | 4.64(1.24) | -8.07(1.62) | - | n.s. | - |
|  |  | Quadratic | 4.10(0.19) | | 3.24(0.40) | -26.47(8.55) | n.s. | - | - |
|  |  | Quadratic | 4.18(0.25) | | n.s. | -31.97(11.23) | n.s. | n.s. | n.s. |
|  | R | **Linear** | **3.38(0.07)** | | **3.17(0.39)** | **-6.26(1.22)** | - | - | - |
|  |  | Linear | 3.37(0.08) | | 3.60(1.23) | -5.88(1.61) | - | n.s. | - |
|  |  | Quadratic | 3.68(0.19) | | 3.19(0.39) | n.s. | n.s. | - | - |
|  |  | Quadratic | 3.70(0.25) | | n.s. | n.s. | n.s. | n.s. | n.s. |
| Putamen | L | **Linear** | **5.66(0.08)** | | **4.99(0.51)** | **-21.61(1.55)** | - | - | - |
|  |  | Linear | 5.60(1.09) | | 6.32(1.59) | -20.37(2.08) | - | n.s. | - |
|  |  | Quadratic | 5.71(0.23) | | 4.99(0.51) | n.s. | n.s. | - | - |
|  |  | Quadratic | 5.62(0.32) | | n.s. | n.s. | n.s. | n.s. | n.s. |
|  | R | Linear | 5.41(0.07) | | 4.70(0.43) | -20.94(1.32) | - | - | - |
|  |  | **Linear** | **5.25(0.09)** | | **7.64(1.35)** | **-17.82(1.76)** | **-** | **-5.89(2.64)** | - |
|  |  | Quadratic | 5.57(0.20) | | 4.70(0.43) | -28.20(8.66) | n.s. | - | - |
|  |  | Quadratic | 5.62(0.27) | | n.s. | -34.87(11.72) | n.s. | n.s. | n.s. |
| Accumbens | L | **Linear** | **0.63(0.01)** | | **0.33(0.08)** | **-3.85(0.25)** | - | - | - |
|  |  | Linear | 0.63(0.02) | | n.s. | -3.73(0.33) | - | n.s. |  |
|  |  | Quadratic | 0.62(0.04) | | 0.34(0.08) | n.s. | n.s. | - | - |
|  |  | Quadratic | 0.59(0.05) | | n.s. | n.s. | n.s. | n.s. | n.s. |
|  | R | **Linear** | **0.61(0.01)** | | **0.49(0.08)** | **-2.87(0.24)** | - | - | - |
|  |  | Linear | 0.59(0.02) | | 0.73(0.24) | -2.58(0.31) | - | n.s. | - |
|  |  | Quadratic | 0.64(0.03) | | 0.47(0.08) | -4.46(1.52) | n.s. | - | - |
|  |  | Quadratic | 0.68(0.05) | | n.s. | -6.54(2.06) | n.s. | n.s. | n.s. |
| Pallidum | L | **Linear** | **1.52(0.03)** | | **1.55(0.18)** | **-3.86(0.55)** | - | - | - |
|  |  | Linear | 1.47(0.04) | | 2.70(0.56) | -2.79(0.73) | - | n.s. | - |
|  |  | Quadratic | 1.69(0.08) | | 1.54(0.18) | -11.71(3.57) | n.s. | - | - |
|  |  | Quadratic | 1.72(0.11) | | n.s. | -14.46(4.84) | 1.20(0.49) | n.s. | n.s. |
|  | R | Linear | 1.53(0.02) | | 1.55(0.15) | -3.68(0.45) | - | - | - |
|  |  | **Linear** | **1.48(0.03)** | | **2.75(0.46)** | **-2.58(0.60)** | - | **-2.47(0.89)** | - |
|  |  | Quadratic | 1.63(0.07) | | 1.55(0.15) | -8.31(2.93) | n.s. | - | - |
|  |  | Quadratic | 1.66(0.09) | | n.s. | -10.98(3.96) | n.s. | n.s. | n.s. |
| Thalamus | L | Linear | 9.07(0.11) | | 8.89(0.69) | -31.79(2.09) | - | - | - |
|  |  | Linear | 8.91(0.15) | | 12.29(2.14) | -28.66(2.80) | - | n.s. | - |
|  |  | **Quadratic** | **8.07(0.34)** | | **8.95(0.69)** | **n.s.** | **-4.93(1.54)** | - | - |
|  |  | Quadratic | 8.93(0.42) | | n.s. | n.s. | n.s. | n.s. | n.s. |
|  | R | Linear | 7.92(0.10) | | 6.23(0.59) | -25.56(1.77) | - | - | - |
|  |  | **Linear** | **7.70(0.12)** | | **10.96(1.80)** | **-21.20(2.36)** | - | **-9.82(3.54)** | - |
|  |  | Quadratic | 7.66(0.27) | | 6.23(0.59) | n.s. | n.s. | - | - |
|  |  | Quadratic | 8.23(0.35) | | n.s. | -46.02(15.54) | n.s. | n.s. | n.s. |
| Hippocampus | L | Linear | 4.68(0.06) | | 3.17(0.04) | -12.84(1.14) | - | - | - |
|  |  | Linear | 4.56(0.08) | | 5.78(1.16) | -10.43(1.52) | - | n.s. |  |
|  |  | **Quadratic** | **3.85(0.17)** | | **3.18(0.37)** | **25.27(7.28)** | **-3.90(0.74)** | - | - |
|  |  | Quadratic | 4.21(0.22) | | n.s. | n.s. | n.s. | n.s. | n.s. |
|  | R | Linear | 4.66(0.06) | | 2.63(0.36) | -11.63(1.10) | - | - | - |
|  |  | Linear | 4.50(0.08) | | 6.14(1.12) | -8.39(1.47) | - | -7.28(2.20) | - |
|  |  | **Quadratic** | **3.96(0.16)** | | **2.64(0.36)** | **20.63(7.10)** | **-3.30(0.72)** | - | - |
|  |  | Quadratic | 4.32(0.22) | | n.s. | n.s. | n.s. | n.s. | n.s. |
| Amygdala | L | Linear | 1.58(0.03) | | 1.50(0.15) | -4.33(0.46) | - | - | - |
|  |  | Linear | 1.53(0.03) | | 2.53(0.47) | -3.38(0.62) | - | n.s. | - |
|  |  | **Quadratic** | **1.38(0.07)** | | **1.50(0.15)** | n.s. | **-0.91(0.31)** | - | - |
|  |  | Quadratic | 1.40(0.09) | | n.s. | n.s. | n.s. | n.s. | n.s. |
|  | R | Linear | 1.62(0.03) | | 1.55(0.15) | -3.97(0.46) | - | - | - |
|  |  | Linear | 1.58(0.03) | | 2.52(0.48) | -3.08(0.62) | - | n.s. | - |
|  |  | **Quadratic** | **1.43(0.07)** | | **1.55(0.15)** | n.s. | **-0.88(0.31)** | - | - |
|  |  | Quadratic | 1.52(0.09) | | n.s. | n.s. | n.s. | n.s. | n.s. |

^a^ Intercept is the extrapolated value at age zero.

– = not applicable; n.s. = non-significant; HS = Hemisphere; L = left; R = Right.

*p* <0.05, Bonferroni corrected with thresholds of 0.015 volumes

In bold font: best fit model

Table S5. Fitting parameters for asymmetries of subcortical structures versus age

|  |  | Parameter(S.E.) | | | | | |
| --- | --- | --- | --- | --- | --- | --- | --- |
| Measures | Fit model | Intept^a^  (×10) | Sex | Age(×10^-1^) | Age^2^  (×10^-2^) | age×sex (×10^-1^) | age^2^×sex (×10^-2^) |
| Caudate | **Linear** | **1.06(0.12)** | **n.s.** | **-0.83(0.21)** | - | - | - |
|  | Linear | 0.94(0.15) | n.s. | n.s. | - | n.s. | - |
|  | Quadratic | 1.27(0.32) | n.s. | n.s. | n.s. | - | - |
|  | Quadratic | 1.46(0.43) | n.s. | n.s. | n.s. | n.s. | n.s. |
| Putamen | Linear | 4.80(0.10) | n.s. | n.s. | - | - | - |
|  | Linear | 0.63(0.13) | n.s. | n.s. | - | n.s. | - |
|  | Quadratic | n.s. | n.s. | n.s. | n.s. | - | - |
|  | Quadratic | n.s. | n.s. | n.s. | n.s. | n.s. | n.s. |
| Accumbens | Linear | 8.11(2.64)_ | n.s. | -2.81(0.48) | - | - | - |
|  | Linear | 1.54(0.34) | n.s. | -3.29(0.97) | - | n.s. | - |
|  | Quadratic | n.s. | n.s. | n.s. | n.s. | - | - |
|  | **Quadratic** | **-2.59(0.97)** | n.s. | **13.58(4.25)** | **-1.73(0.43)** | **-18.50(6.27)** | **2.01(0.63)** |
| Pallidum | Linear | n.s. | n.s. | n.s. | - | - | - |
|  | Linear | n.s. | n.s. | n.s. | - | n.s. | - |
|  | **Quadratic** | n.s. | n.s. | **-6.83 (2.34)** | **0.75(0.24)** | - | - |
|  | Quadratic | n.s. | n.s. | n.s. | 0.73(0.29) | n.s. | n.s. |
| Thalamus | Linear | 1.33(0.83) | 1.89(0.50) | n.s. | - | - | - |
|  | Linear | 1.40(0.11) | n.s. | n.s. | - | n.s. | - |
|  | Quadratic | 0.94(0.23) | 1.89(0.50) | n.s. | n.s. | - | - |
|  | Quadratic | 0.95((0.31)) | n.s. | n.s. | n.s. | n.s. | n.s. |
| Hippocampus | Linear | n.s. | n.s. | n.s. | - | - | - |
|  | **Linear** | n.s. | n.s. | **-0.53(0.22)** | - | **0.85(0.33)** | - |
|  | Quadratic | n.s. | n.s. | n.s. | n.s. | - | - |
|  | Quadratic | n.s. | n.s. | n.s. | n.s. | n.s. | n.s. |
| Amygdala | Linear | n.s. | n.s. | n.s. | - | - | - |
|  | Linear | n.s. | n.s. | n.s. | - | n.s. | - |
|  | Quadratic | n.s. | n.s. | n.s. | n.s. | - | - |
|  | Quadratic | n.s. | n.s. | n.s. | n.s. | n.s. | n.s. |

^a^ Intercept is the extrapolated value at age zero. – = not applicable; n.s. = non-significant

*p* <0.05, Bonferroni corrected with thresholds of 0.013 volumes

In bold font: best fit model
